# Transverse Kerker effect in all-dielectric spheroidal particles

**DOI:** 10.1038/s41598-022-11733-4

**Published:** 2022-05-14

**Authors:** Mikhail M. Bukharin, Vladimir Ya. Pecherkin, Anar K. Ospanova, Vladimir B. Il’in, Leonid M. Vasilyak, Alexey A. Basharin, Boris Luk‘yanchuk

**Affiliations:** 1 National University of Science and Technology “MISiS”, Moscow, 119049 Russia; 2 Joint Institute for High Temperatures, Russian Academy of Sciences, Moscow, 125412 Russia; 3 Department of Physics and Mathematics, Institute of Photonics, University of Eastern Finland, Joensuu, 80101 Finland; 4 Dept. Math. Mechan., St. Petersburg State University, St. Petersburg, 198504 Russia; 5 Petersburg University of Aerospace Instrumentation, St. Petersburg, 190000 Russia; 6 Main (Pulkovo) Astronomical Observatory of RAS, St. Petersburg, 196140 Russia; 7 Institute for Theoretical and Applied Electromagnetics RAS, Moscow, 125412 Russia; 8 Faculty of Physics, Lomonosov Moscow State University, Moscow, 119991 Russia

**Keywords:** Optics and photonics, Optical materials and structures, Microresonators

## Abstract

Kerker effect is one of the unique phenomena in modern electrodynamics. Due to overlapping of electric and magnetic dipole moments, all-dielectric particles can be invisible in forward or backward directions. In our paper we propose new conditions between resonantly excited electric dipole and magnetic quadrupole in ceramic high index spheroidal particles for demonstrating transverse Kerker effect. Moreover, we perform proof-of-concept microwave experiment and demonstrate dumbbell radiation pattern with suppressed scattering in both forward and backward directions and enhanced scattering in lateral directions. Our concept is promising for future planar lasers, nonreflected metasurface and laterally excited waveguides and nanoantennas.

## Introduction

Recently, the studies of light manipulation in electromagnetic structures have emerged as promising scientific fields due to unusual multipoles interactions in subwavelength all-dielectric and plasmonic particles^1^. The transition from electronic systems and information processing methods to optical ones strongly requires development of high Q-factor open resonators and nanoantennas enabling effective light controlling at the nanoscale in optical microcircuits^2^. However, an implementation of strongly resonance nanoparticles requires complex geometries at nanoscale and forces to search for qualitatively new solutions^3^.

In this issue, all-dielectric photonics brings the idea of resonant scattering on dielectric particles based on the so-called Mie resonances^4^. Firstly introduced for spherical particles, its definition was extended to particles of various shapes and based on electromagnetic multipoles interactions. There is a plenty of research on dielectric particles of different geometries demonstrating strong fields localizations, Fano-resonances^5^, Kerker conditions^6^, anapole modes^7^, invisible metasurfaces^8^ and Bound states in the continuum (BIC)^9^.

Primarily, invisible physics inspired us to study multipolar interference between multipoles of so-called trivial and nontrivial families^6^. For instance first and second Kerker effects are results of interaction between electric and magnetic dipole moments fulfilling condition for zero backward or forward scattering^10,11^. Similarly, Kerker effect can be observed in interaction between quadrupoles of electric and magnetic types^6^. For the first time, Kerker effect was experimentally demonstrated for ceramic sphere in microwave^12^ and then for silicon nanosphere^13^ and gallium arsenide nano-disks in optics^14^. The main problem for demonstrating Kerker effects in all-dielectric particles is overlapping between resonances of electric and magnetic dipole moments in the same frequency^15^. For example, the magnetic and electric dipole resonances are separated from each other for silicon nanosphere. The solution can be found in the application of spheroidal particles giving optimum aspect ratio for overlapping electric and magnetic resonances provide suppressed backward scattering and strong forward scattering^16^.

The second level of Kerker-scattering can be defined as suppression of radiation in both forward and backward directions and named as *generalized Kerker effect*^6,17^. In this issue, the particles of disks, cuboids shape^18^ and onions multilayer particles^17^ can be considered invisible due to interactions between electric and magnetic dipoles as well as between their quadrupoles. Moreover, metasurfaces based on them are almost transparent and unaccompanied by phase change with light transmission^8^. From practical applications, simultaneous excitation of Kerker’s first and second conditions becomes crucially important for strong near-field localization and developing of nonreflected metasurfaces for advanced photonic technologies.

For the first glance, generalized Kerker effect may seem similar to electric and magnetic anapole states and hybrid anapole as well^19–21^. However, the main difference is that generalized Kerker effect is accompanied by transverse scattering while anapole particle does not scatter at all exceptionally up to higher multipoles in agreement with optical theorem^22–26^. On the other hand, the side scattering property can be usefully exploited for metasurfaces as a platform for planar lasers via transverse Kerker effect as coupling between near fields of particles and for lateral excitation of nano-waveguides^2,8,18^.

For this aim, we theoretically propose and demonstrate via proof-of-concept microwave experiment an intensive lateral scattering by spheroidal all-dielectric particle. We demonstrate this effect on the spheroidal particle with the aspect ratio close to 2.1 between resonantly coincidental electric dipole and magnetic quadrupole moments which gives transverse radiation pattern of dumbbell form in the selected direction perpendicular to the incident wave front. Experimental results match theoretical results. We should also note that we mean “proof-of-concept” definition for our experiment in order to show transverse Kerker effect, which can be demonstrated also for other shapes of meta-particles and permittivities.

We have previously implemented a number of studies to suppress scattering in an elliptical particle^21^ due to hybrid anapole and this work is continuation of these studies. The novelty of the theoretical part of our paper is the optimization of the transverse Kerker effect by choosing the optimal shape of a spheroidal all-dielectric particle. This work originated from a previously published paper^15^ which explored the possibility of maximizing forward scattering while completely suppressing backward scattering by choosing the shape of a spheroid particle. Moreover, In our work, we analyzed for the first time the possibility of maximizing the transverse Kerker effect for a spheroidal particle. In this paper, we theoretically involve the interaction between electric dipole and magnetic quadrupole moments. We find the formulas for a description of the interference conditions needed for longitudinal and transverse dumbbell scattering. These conditions describe relative amplitude and phases of electric dipole and magnetic quadrupole excited in spheroidal particles.

To confirm the proposed transverse Kerker effect origin of dumbbell scattering, we experimentally observe for the first time the radiation pattern of spheroidal particle in the microwave regime.

We note that the study of electromagnetic response of spheroidal particles is significant by several reasons. Dust grains in the interplanetary and interstellar medium are assumed to have spheroidal shape^27^, and their response may be explained by our approach. Moreover, spheroidal particles are equally important for the problem of atmosphere optics^28^, medicine and microbiology^29^.

Additionally, resonant scattering phenomena have been demonstrated in another nonspherical particles. Nonradiating mode conditioned by so-called hybrid anapole establishment demonstrated in high-index dielectric ellipsoidal particles^21^ and in all-dielectric nanocylinders^30^.

## Dumbbell radiation due to multipoles interaction

We start with finding of the transverse scattering conditions from the multipole decomposition of the field radiated by the arbitrary particle^31^. We note that transverse Kerker effect is interpreted as suppression of both scattering in forward and backward directions providing enhanced scattering in lateral directions^18^.

In our consideration, we use only families of dipoles and quadrupoles.

The radiation of arbitrary source is formulated by electric field of multipoles. For *l*=1, radiation is presented by electric and magnetic dipoles, while for *l*=2 by electric and magnetic quadrupoles:

1 $$\begin{aligned}&\mathbf{E}_{(total)} (\theta , \varphi , r) \approx \mathbf{E}_{(l=1)} +\mathbf{E}_{(l=2)} , \nonumber \\&\quad \mathbf{E}_{(l=1)} (\theta ,\varphi ,r)\approx \frac{\mu _{0} c^{2} }{3\sqrt{2\pi } } \frac{\exp (-ikr)}{r} \sum _{m=0,\pm 1}\left[ \left( k^{2} Q_{1,m} -ik^{3} T_{1,m} \right) \right. \nonumber \\&\left. \qquad \times \left( \mathbf{Y}_{1,2,m} +\sqrt{2} \mathbf{Y}_{1,0,m} \right) +i\sqrt{3} \left( k^{2} M_{1,m} \times \mathbf{Y}_{1,1,m} \right) \right] , \nonumber \\&\quad \mathbf{E}_{(l=2)} (\theta ,\varphi ,r)\approx \frac{\mu _{0} c^{2} }{10\sqrt{6\pi } } \frac{\exp (-ikr)}{r} \sum _{m=0,\pm 1,\pm 2}\left[ ik^{3} Q_{2,m}^{(e)} \times (\sqrt{2} \mathbf{Y}_{2,3,m} +\sqrt{3} \mathbf{Y}_{2,1,m} )\right. \nonumber \\&\left. \qquad -\sqrt{5} k^{3} Q_{2,m}^{(m)} \times \mathbf{Y}_{2,2,m} \right] . \end{aligned}$$

Here, $\mu {}_{0}$ is the magnetic permeability of vacuum, *c* is the speed of light, *r* is the radius-vector, and **Y** ${}_{k,l,m}$ are the spherical vector harmonics. The spherical multipoles are related to the Cartesian multipoles, that is, electric dipole ***p***, magnetic dipole ***M***, toroidal dipole moment ***T***, electric quadrupole *Qe*, magnetic quadrupole *Qm*, as follows:

$$\begin{aligned} Q_{1,0}= &\,  p_{z}, \ \ \ \ Q_{1,\pm 1} =\frac{{\mp } p_{x} +ip_{y}}{\sqrt{2}}, \ \ \ \ T_{1,0} = T_{z},\ \ \ \ T_{1,\pm 1} =\frac{{\mp } T_{x} +iT_{y}}{\sqrt{2}}, \ \ \ \ M_{1,0} =-M_{z}, \ \ \ \ M_{1,\pm 1} =\frac{\pm M_{x} -iM_{y}}{\sqrt{2}} , \\ Qe_{2,0}=\, & 3\,Qe_{zz}, \ \ \ \ {Qe_{2,\pm 1}^{} = \sqrt{6}\, \left( {\mp } Qe_{xz}^{} +iQe_{yz}^{} \right) }, \ \ \ \ {Qe_{2,\pm 2}^{} = \frac{\sqrt{6} }{2} \,\left( Qe_{xx}^{} {\mp } i2Qe_{xy}^{} -Qe_{yy}^{} \right) }, \\ Qm_{2,0}^{}= & \,{} -\frac{3}{2}\, Qm_{zz}^{} , \ \ \ \ {Qm_{2,\pm 1}^{} =\sqrt{\frac{3}{2} } \,(\pm Qm_{xz}^{} -iQm_{yz}^{} )}, \ \ \ \ {Qm_{2,\pm 2}^{} =\frac{\sqrt{6} }{4}\, (-Qm_{xx}^{} \pm i2Qm_{xy}^{} +Qm_{yy}^{} )}. \end{aligned}$$

Cartesian multipoles are calculated by integrating over the current density ***J****(****r****)* distribution within the particle volume and $\alpha$,$\beta$,$\gamma$ = *x,y,z*:

$$\begin{aligned} { p}_{\alpha }= & {} \frac{1}{i\omega } \int { J}_{\alpha } d^{3} { r}, \ \ \ \ { m}_{\alpha } =\frac{1}{2c} \int \left[ { r}\times { J}\right] _{\alpha } d^{3} { r}, \ \ \ \ T_{\alpha } =\frac{1}{10c} \int \left[ { (r}\cdot { J)}{} { r}_{\alpha } { -2r}^{2} { J}_{\alpha } \right] d^{3} r, \ \ \ \ P_{\alpha } =-p_{\alpha } +ikT_{\alpha }, \\ Qe_{\alpha \beta }= & {} \frac{1}{i2\omega } \int \left[ { r}_{\beta } { J}_{\alpha } +{ r}_{\alpha } { J}_{\beta } -\frac{2}{3} ({ r}\cdot { J})\delta _{\alpha \beta } \right] d^{3} { r}, \ \ \ \ Qm_{\alpha \beta } =\frac{1}{3c} \int \left[ { r}_{\alpha } \left[ { r}\times { J}\right] _{\beta } +{ r}_{\beta } \left[ { r}\times { J}\right] _{\alpha } \right] d^{3} { r}. \end{aligned}$$

Here $\delta {}_{\alpha \beta }$ is the Kronecker symbol.

The scattering cross section of the particle can be described by terms of multipoles:

2 $$\begin{aligned} \sigma _{scat} =\frac{k^{4} }{6\pi \varepsilon _{0}^{2} |E_{0} |} \left( |{ M}|^{2} +|{ P}|^{2} \right) +\frac{k^{6} }{80\pi \varepsilon _{0}^{2} |E_{0} |} \left( 4|Qe_{\alpha \beta }^{} |^{2} +|Qm_{\alpha \beta }^{} |^{2} \right) . \end{aligned}$$

Let us suppose two transverse scattering cases, mutually perpendicular to each other:

1. $\theta$ = 0 and $\theta$ = $\pi$. In this case multipoles radiate only in transverse to incident wave direction and scattering fields ***E*** ${}_{(total)}$($\theta$=0) = 0 and ***E*** ${}_{(total)}$($\theta$=$\pi$) = 0.
2. $\theta$ = $\pi$/2 and $\theta$ = 3$\pi$/2. In this case multipoles radiate only along incident wave direction and scattering fields ***E*** ${}_{(total)}$($\theta$=$\pi$/2) = 0 and ***E*** ${}_{(total)}$($\theta$=3$\pi$/2) = 0.

From Eq. (1) one can simply find solution for conditions 1 and 2 (see “Methods”), and we get relations between electric dipole ***P*** and magnetic quadrupole *Q*${}_{m}$ . However, we imply other multipoles have insufficient response in our system:

3 $$\begin{aligned} P_{\alpha }= & {} -\frac{ik}{2} Qm_{\beta \gamma } , \end{aligned}$$

4 $$\begin{aligned} P_{\alpha }= & {} \frac{ik}{2} Qm_{\beta \gamma } . \end{aligned}$$

The same conditions can be obtained for magnetic dipole and electric quadrupole.

This simple result reveals that zero radiation (Transverse Kerker effect) simultaneously in backward and forward directions, Condition 1, Eq. (3) or simultaneously in lateral directions (Longitudinal Kerker effect) Condition 2, Eq. (4) can be achievable just by interference between two multipoles of electric dipole moment and magnetic quadrupole. Moreover, the radiation direction is almost defined by phase of magnetic quadrupole which indicated by its sign. Similar results were obtained numerically by Asano & Yamamoto^32^, but without explaining the reason for side scattering.

Recently, Jeng Yi Lee et al.^17^ formulated conditions for simultaneously nearly zero forward and backward scattering. It is possible when the first (dipoles) and second (quadrupoles) order multipoles excited in particle obey the condition $a_{1}=-5/3\,b_{2}$ and $b_{1}=-5/3\,a_{2}$. However, for simple all-dielectric sphere the coexistence of electric and magnetic dipoles and their quadrupoles is impossible at the same frequencies. Then, the multilayer structures^17^, spherical particles with radial anisotropy give chance for experimental evidence of transverse Kerker effect^33^.

**Figure 1:**
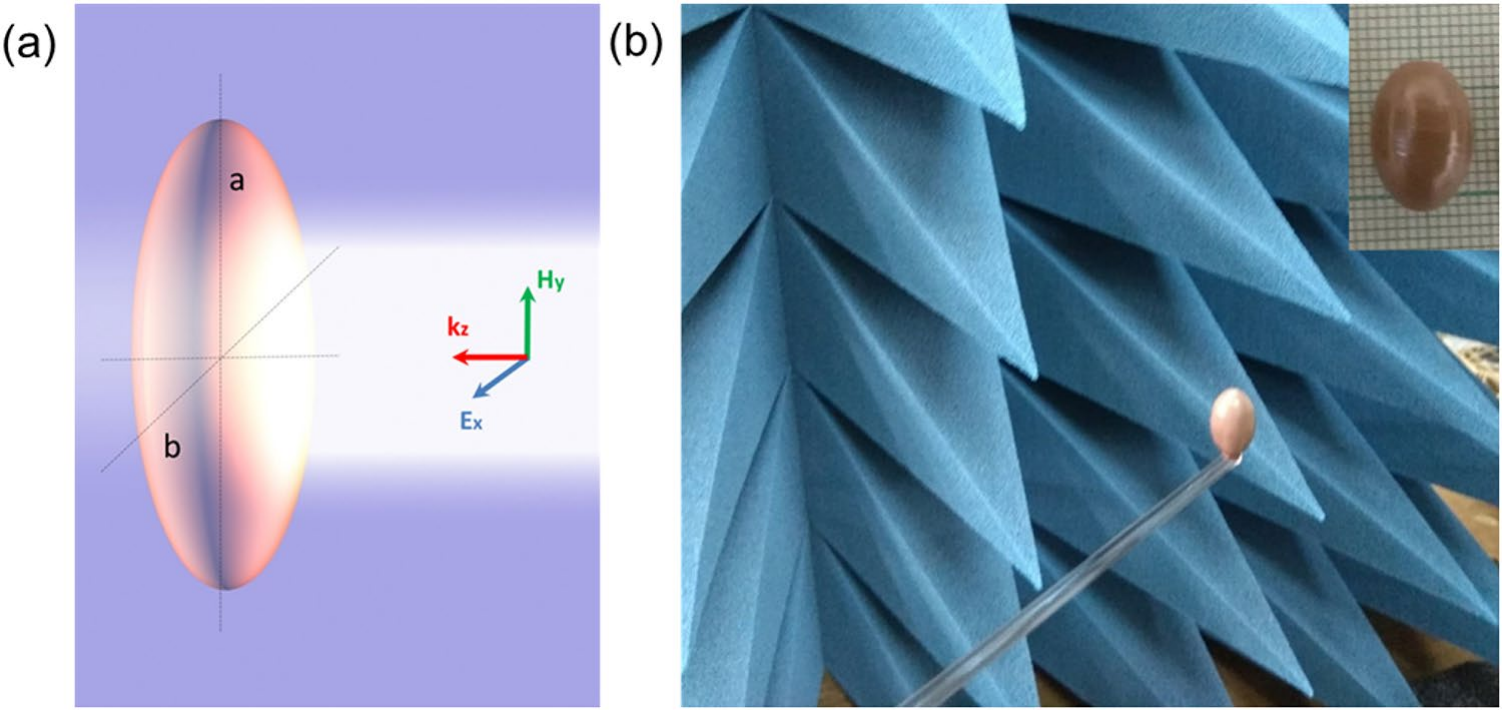
(**a**) Illustration of high-index dielectric prolate spheroidal particle with dielectric permittivity $\varepsilon$=150 and the major and minor semiaxes of *a* and *b*, respectively. Linearly polarized plane wave is impinging laterally with vector ***E*** parallel to minor axis. (**b**) Experimental sample of ceramic spheroidal particle with *a* = 19.5/2 mm and *b* = 12.5/2 mm in anechoic chamber. The inset shows picture of sample.

To realize this unique property, let us consider the electromagnetic scattering by high-index dielectric spheroidal particle with *a* and *b* being the major and minor semiaxis, respectively. The aspect ratio *a/b* indicates the spheroidal shape changing from needle (*a/b* > 1, prolate spheroid), passing by sphere (*a/b*=1), to disk (*a/b* < 1, oblate spheroid). During our experimental study, we suppose the case of prolate spheroid with *a* = 19.5/2 mm and *b* = 12.5/2 mm, and ratio *a/b* = 1.56. The dielectric permittivity of particle is $\varepsilon$ = 150 that presented as high-index ceramics. The particle is illuminated by plane wave at lateral incidence with polarization of vector ***E*** parallel to the minor spheroidal axis *x*. The particle geometry and excited wave are depicted in Fig. 1.

In our paper, we demonstrate spheroidal all-dielectric particle as promising for demonstration of transverse Kerker effect due to extra radius as a channel for multipoles tunability in comparison with sphere.

For this, we consider the spheroid with shape given by equation:

5 $$\begin{aligned} \frac{x^{2} }{b^{2} } +\frac{y^{2} }{a^{2} } +\frac{z^{2} }{b^{2} } =1 \end{aligned}$$

**Figure 2:**
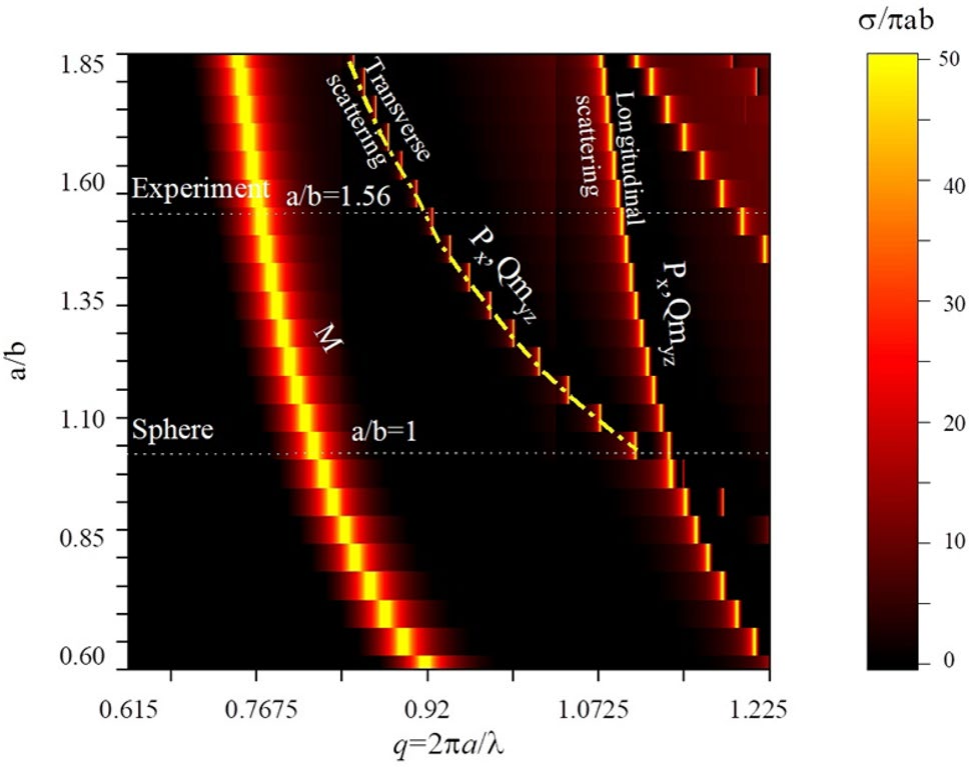
Normalized scattering cross-section of spheroidal all-dielectric particles depending on their aspect ratio *a/b* and size parameter $q=2\pi a/\lambda$. Experimental cross-section obtained for *a/b*=1.56 demonstrates three peaks, two of them are related to transverse and longitudinal scatterings.

In order to demonstrate transverse scattering approach, we perform simulation of electromagnetic scattering by spheroidal particles of different ratio *a/b* (Fig. 2). We use commercial version of CST Microwave Studio and Time domain solver with open boundary conditions. The particle is illuminated by a plane wave with linearly polarized ***E*** component (Fig. 1). The scattering cross section $\sigma$ is normalized to $\sigma$/$\pi$*ab*. For small ratio *a/b* < 1, the particle is of disk shape and its scattering spectrum is defined by two main resonances where magnetic dipole moment *M* is the first term and electric dipole *P* is the second one. This trend is not changing up to *a/b* = 1, i.e. transition of disk to the sphere. We observe that the bifurcation point *a/b* = 1 and *q* = 1.1 is splitting into two resonances for *a/b* > 1. Both of them are characterized by electric dipole moment and magnetic quadrupole moment. However, the main difference between them is determined in the phases of the magnetic quadrupoles. For the first case (Transverse scattering), electric dipole moment *P*${}_{x}$ and magnetic quadrupole *Qm*${}_{yz}$ components have positive sign of phases, while the second resonance (Longitudinal scattering) is characterized by the opposite sign of phases (Fig. 2).

**Figure 3:**
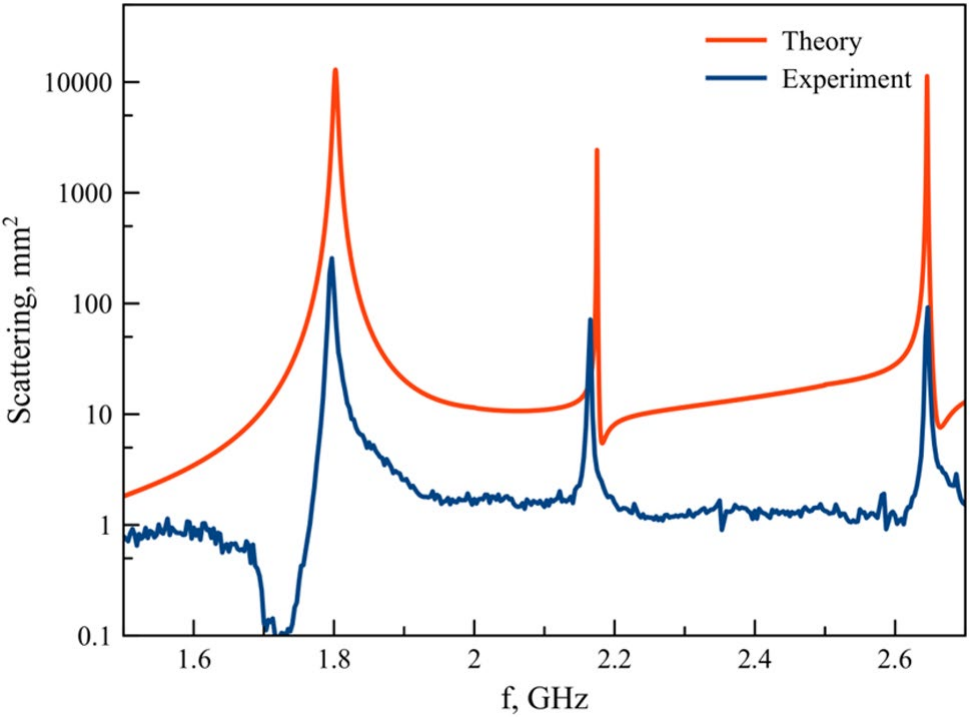
Simulated and measured scattering characteristics of spheroid with aspect ratio *a/b* = 1.56 in frequency range 1.5–2.5 GHz.

To propose features of electromagnetic response of prolate spheroid (*a/b* > 1) and their modes, we consider the spectra calculated by CST Microwave Studio of particle with *a/b*=1.56 and demonstrate its experimental scattering cross-section (Fig. 3). The resonance peeks on 1.8 GHz, 2.17 GHz and 2.65 GHz demonstrate good agreement between theory and experiment.

**Figure 4:**
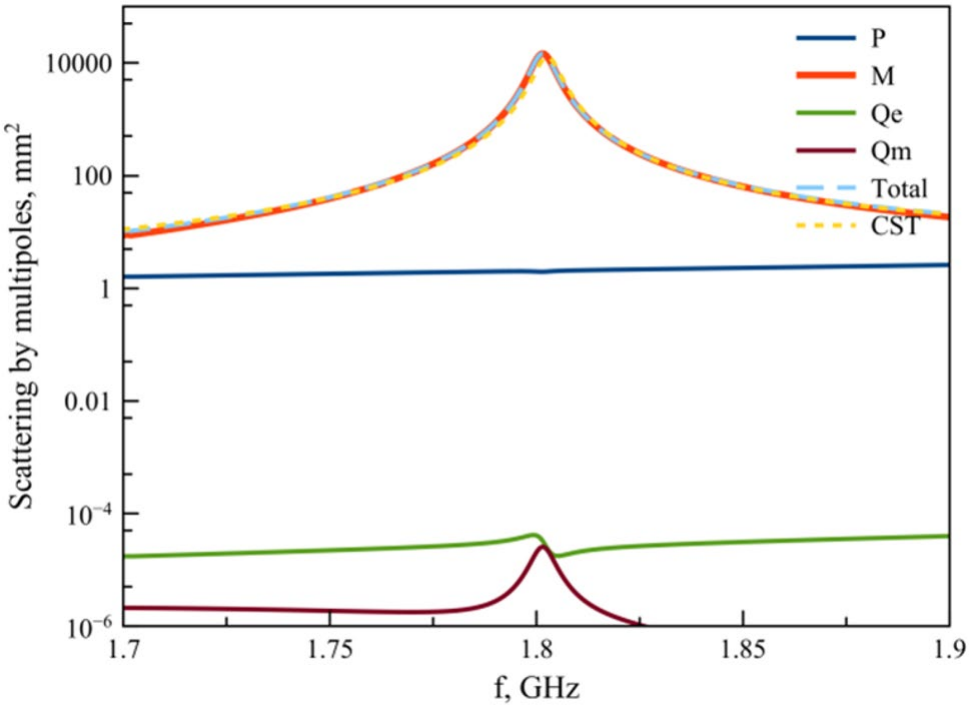
Scattering cross section of multipoles on 1.8 GHz.

In order to clearly understand the origin of peaks, we perform multipole decomposition of four main multipoles excited in system, where ***P*** means electric dipoles, ***M*** magnetic ones, *Qe* electric quadrupoles and *Qm* magnetic ones for first and second resonances. The first peak close to 1.8 GHz is almost defined by magnetic response ***M*** (Fig. 4). Other multipoles are sufficiently suppressed and do not contribute to the response of the system. We also recalculated the shape of the scattering resonance from multipoles, which in good agreement with CST calculated scattering cross section.

**Figure 5:**
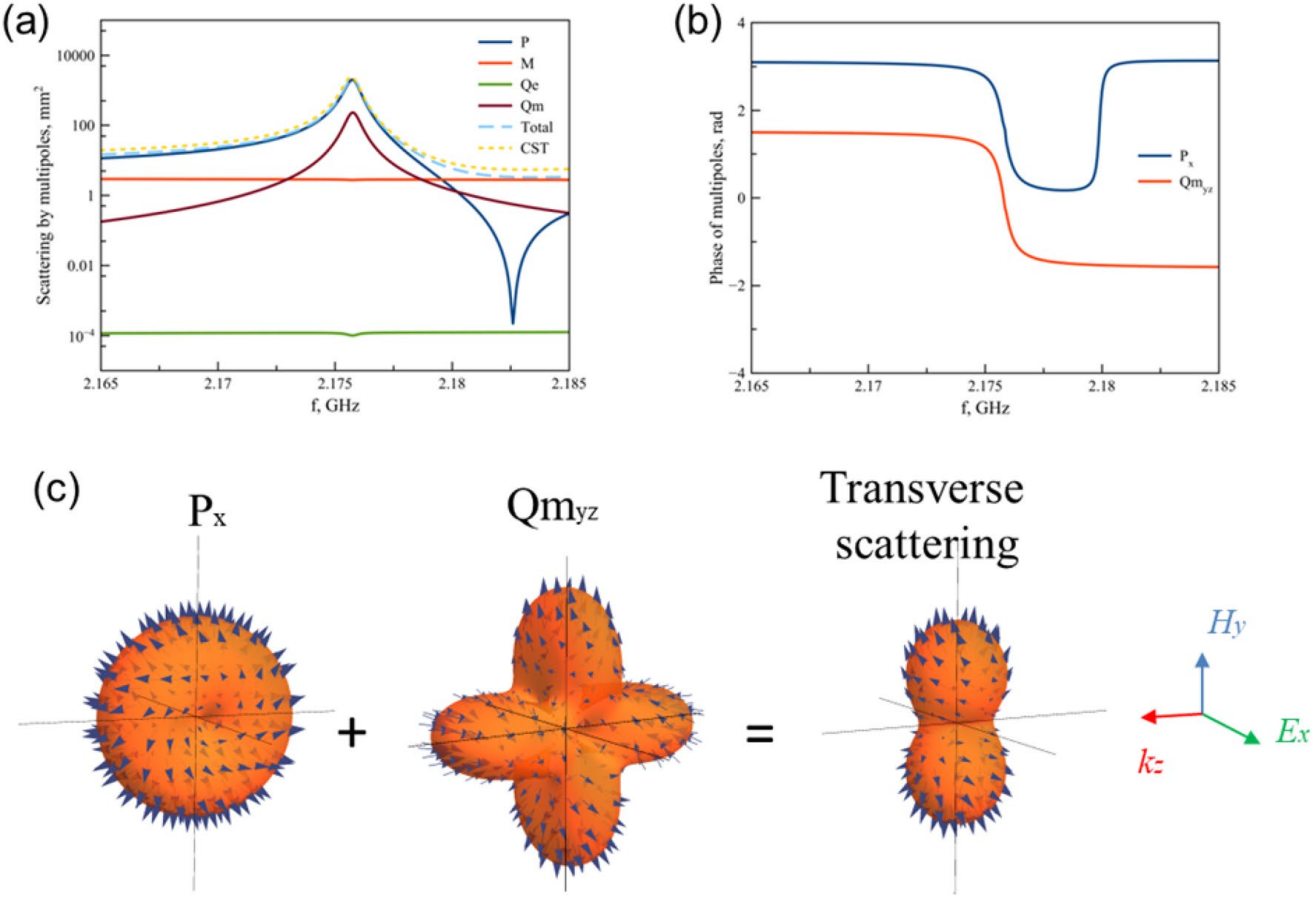
(**a**) Scattering cross section of multipoles on 2.175 GHz. (**b**) Phases of electric dipole and magnetic quadrupole. (**c**) Radiation pattern of excited electric dipole and magnetic quadrupole and total radiation pattern.

The second resonance at 2.17 GHz is a result of interaction between components of electric dipole $P{}_{x}$ and magnetic quadrupole *Qm*${}_{yz}$= *Qm*${}_{zy}$ (Fig. 5). Other multipoles tend to zero and do not contribute to the response of the system.

**Figure 6:**
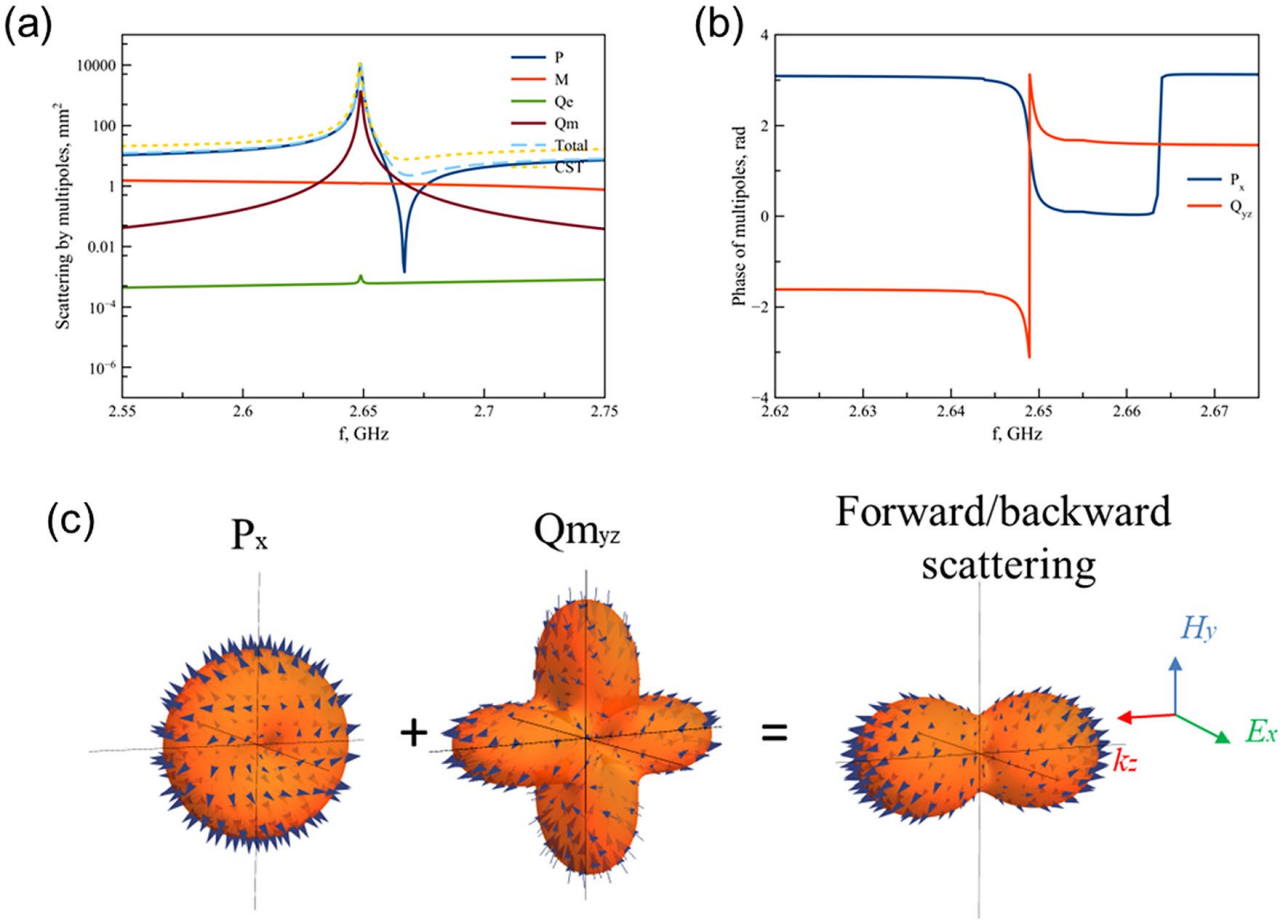
(**a**) Scattering cross section of multipoles on 2.64 GHz. (**b**) Phases of electric dipole and magnetic quadrupole. (**c**) Radiation pattern of excited electric dipole and magnetic quadrupole and total radiation pattern.

The third peak at 2.65 GHz is also characterized by electric dipole *P*${}_{x}$ and magnetic quadroupole *Qm*${}_{yz}$ components (Fig. 6). Indeed, scattering responses at 2.17 GHz (Fig. 5a) and 2.65 GHz (Fig. 6a) have identical multipoles contributions. However, we analyze their phases close to resonances in order to find difference between them. The phases of electric dipole and magnetic quadrupole on 2.175 GHz (Fig. 5b) are both positive and equal to 1.752 rad and 0.1338 rad, respectively. However, the phase of magnetic quadrupole on 2.648 GHz is negative, namely –1.9 rad (Fig. 6b). In order to demonstrate resulted radiation pattern of resonance peak 2.175 GHz and 2.648 GHz, we plot radiation patterns of electric dipole and magnetic quadrupole taken into account their amplitudes and phases. For resonance at 2.175 GHz (Fig. 5c), electric dipole and magnetic quadrupole radiate with positive phases, accordingly radiation of their interference enhanced in *y* direction and suppressed along *z* axis. Contrary, radiation on 2.648 GHz (Fig. 6c) formed by destructive interference of electric dipole and magnetic quadrupole in *y* direction, but radiation in *z* direction is identical in forward and backward directions.

Thus, the scattering pattern of all-dielectric spheroidal particle is defined by electric dipole and magnetic quadrupole and the phase signs. Our simulation results are in good agreement with Eq. (3).

## Proof-of-concept microwave experiment

For the observation of transverse scattering via proof-of-concept microwave experiment, we use high-voltage capacitor ceramic K15U-2 that is made of titanate-based ceramics ($\hbox {SrTiO}_3$). For this purpose, we fabricate ceramic spheroidal particle with large axis of $2a = 19. 5 \pm 0.1$mm and small axis $2b = 12. 5 \pm 0.1$ mm from a ceramic cylinder by mechanical grinding process. The dimensions of the particle are determined by a caliper whose measurement accuracy is ±0.1 mm. These kind ceramics provide extremely high dielectric permittivity (up to 180) in microwave with an associated loss tangent in the range $\tan \delta = 10^{-4}-10^{-3}$ (see Refs.^34,35^). In our frequency range, the sample loss tangent is $\tan \delta = 3 \cdot 10^{-4}$ and $\varepsilon = 150$ at a frequency of 1.6–2.6 GHz^34^.

In order to observe transverse scattering we perform experiment and measure radiation pattern at 2.17 GHz in two planes *xy* and *yz*.

We use an anechoic chamber with dimensions of $2\times 2\times 3$ m equipped by ECCOSORB absorbers, two wideband horn antennas ETS-Lingren’s model 3115, Rotary table and Vector Network Analyzer Agilent E5071C ENA. We also use 20 dB gain amplifier to increase the signal-to-noise ratio. Our setup permits to measure signal variation on the scale up to − 80 dB. In order to exclude all errors and noise from measured data, we measure scattering cross-section without sample and with sample^36–38^. The reasons for measurement errors in microwave experiments are range from random internal thermal noise of electronic devices to residual external echoes from obstacles of the range, non ideal absorbers, cables, rotators and etc. (See page 120 in^36^). In order to reduce these errors, the signal scattered from particle was normalized to the signal scattered from the area without particle, pages 147–148^36^. This being the case, the background radiation should be measured in the absence of a scatterer, and then scatterer should be measured in the presence of background radiation. The desired scattering signal from the particle have been extracted from the particle-plus-background signal. This processing is provided as an option of VNA Agilent E5071 C ENA.

**Figure 7:**
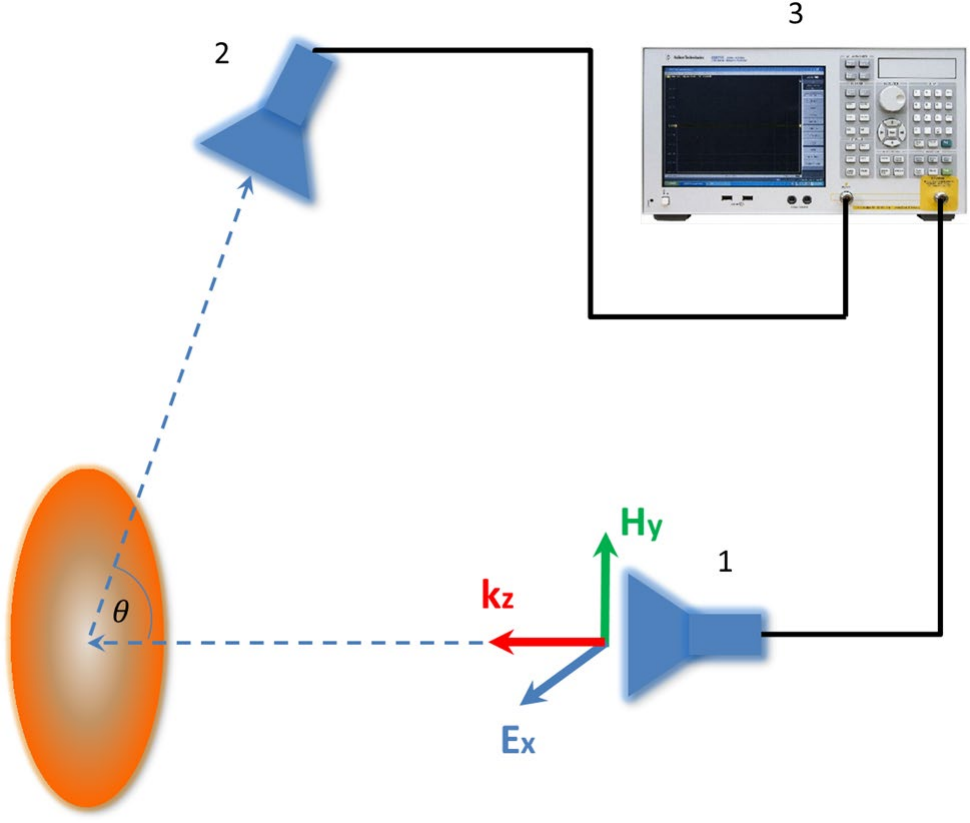
Sketch of experiment setup.1- transmitting antenna, 2- receiving antenna, 3-VNA Agilent E5071 C ENA.

The size of anechoic chamber provides a working zone of about 20 $\hbox {cm}^3$ at frequencies of interest to us, which is quite enough for measuring a spheroidal particle with the size of 20 mm. Considering that broadband horn antennas form a pseudo-spherical front, at a distance greater than the wavelength^36^ within the dimensions of the spheroid, we can consider it as plane wave front, which is sufficient for measuring scattering patterns^36–38^. According to page 130, formula 4.10^36^ far field criterion for measurements is perform: $R>\frac{2d^2}{\lambda }$, where *R*-distance between antenna and particle, *d* -size of particle, $\lambda$- wavelength. The sketch of the experimental setup is shown in Fig. 7. A wide band horn antenna 1 was used to generate the incident wave to excite the spheroidal particle. Another received antenna 2 positioned was used to detect the scattered from the particle signal. The distance between antenna and particle is 1.5 m. The antennas were connected to the ports of a Vector Network Analyzer by a 50 Ohm cable. The polarization of the wave was set as depicted in Fig. 7. In order to measure the radiation pattern we rotated antenna 2 by angle theta around the particle in two planes *xy* and *yz* and measured signal scatterer by the particle.

**Figure 8:**
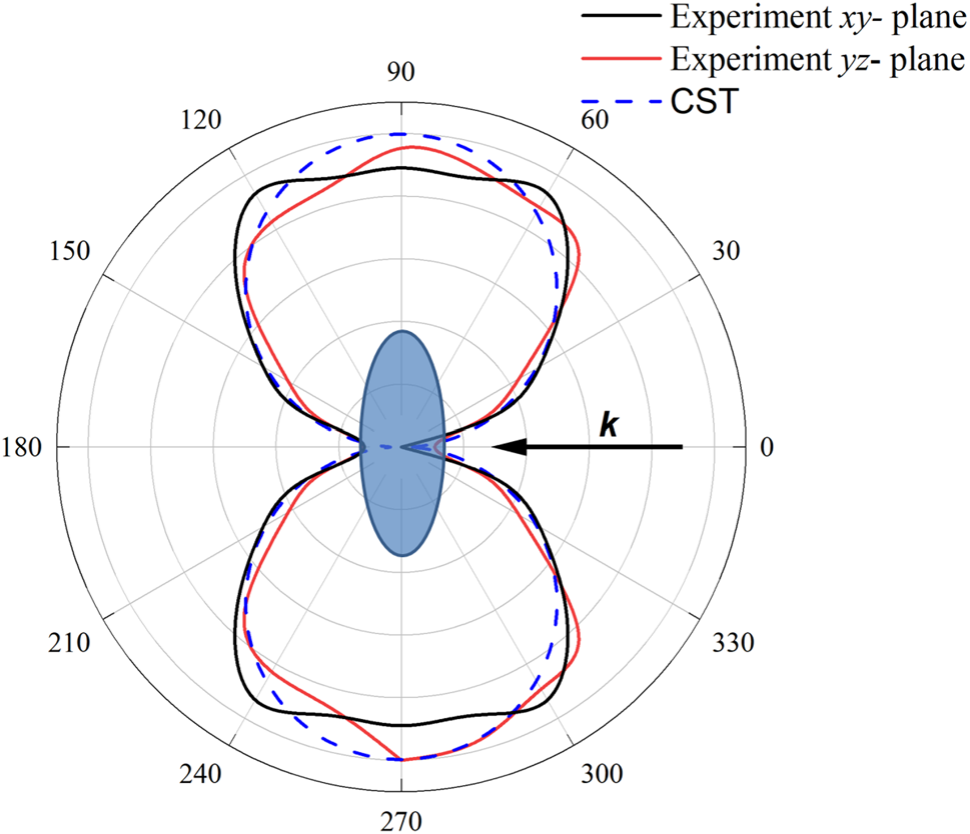
Experimental radiation pattern on 2.17 GHz for *xy-*plane, *yz*-plane compared with theory.

Obviously, the normalized radiation patterns in both planes of spheroid remind the dumbbell shape and coincide with the simulated by CST Microwave Studio pattern (Fig. 8), and we observe strong transverse scattering, reminiscent patterns resulted as interference of electric dipole and magnetic quadrupole multipoles (3) while forward and backward scattering is negligible. We also add theoretical pattern in manner of formula (3) for comparison with theoretical results. Due to our experiment we observe suppression scattering in 0 and 180 deg directions. However, the scattering in lateral 90 and 270 deg directions is pronounced. The distance between emitting antenna and spheroidal particle is 1.5 m. All radiation patterns are in good agreement with simulation results obtained by CST Microwave studio, the measured errors are enough for such kind of anechoic chamber ($\pm 2.5$ dB)^36^. Our results are qualitatively demonstrating dumbbell scattering. However, we suppose that the difference between experimental and CST-calculated curves can be appeared due to presented losses in ceramic material. Thus, the resonant peaks in the experiment (Fig. 3) look less broadened. Moreover, we fabricated spheroids from the cylinders and manufacturing errors lead to the fact that the tops of the spheroids can be more flattened, which gives a wider radiation pattern.

## Summary

Recently, the authors actively discussed the transverse Kerker effect of suppression scattering in forward or backward direction. However, Ufimtsev introduced another principle of invisibility^39^. He showed in 1962 (English translated in 1971) that the waves can bend around of body and diffracted in lateral directions. He called these waves as edges waves. Moreover, our effect of dumbbell-form scattering is different from invisibility effect due to enhanced scattering cross-section, while Kerker effect and Ufimtsev scattering aim to reduce scattering in whole.

We would like to remark that implementation of particles with such dumbbell scattering with near zero forward/backward scattering can be fabricated without complicated anisotropic and multilayer systems, as well as without strong loss plasmonic materials. For a spherical particle, it is impossible to simultaneously suppress forward and backward scattering due to the optical theorem. However, for spheroids and other geometries, the optical theorem is modified to provide suppression of both forward/backward scattering in the presence of strong side scattering. Moreover, we propose that in optical experiments spheroidal particles may be replaced by elliptical cylinders. Note, that transverse dumbbell Kerker effect can be realized in planar structures by interaction of magnetic dipoles and electric quadrupoles.

Moreover, we suppose that in optical experiments spheroidal particles may be fabricated from low-index dielectrics like silicon or silica. The semiconductor nanoparticles behave well in optical range and can be easily tuned for required *nm* dimensions. But their permittivity is not so large ($\varepsilon \sim$16) in optics.

**Figure 9:**
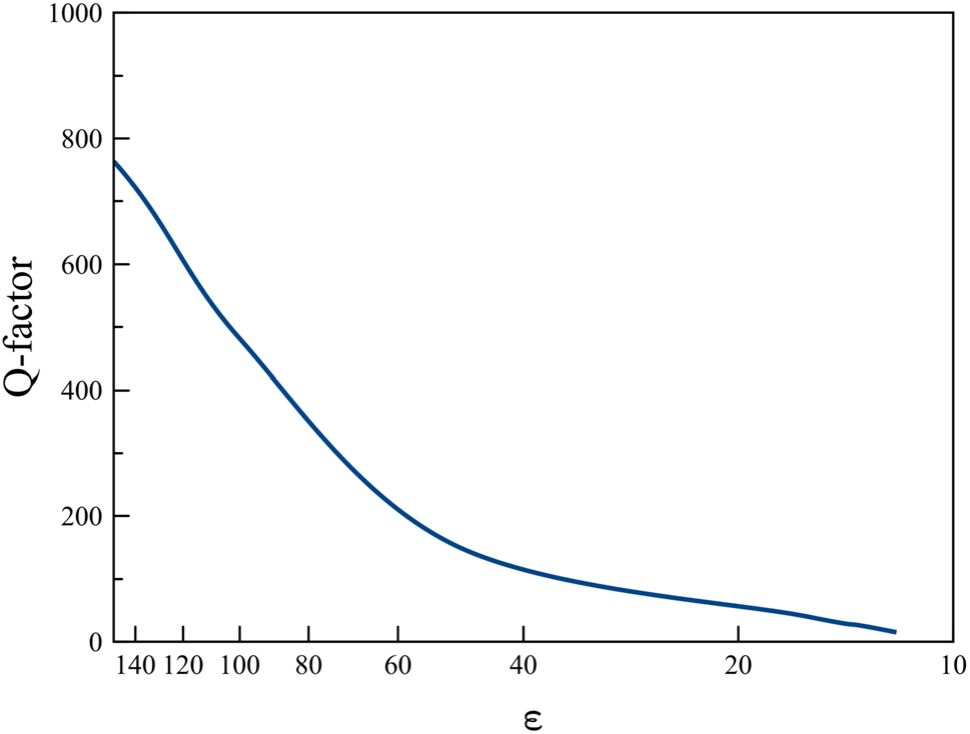
Dependence of dumbbell Kerker effect Q-factor on permittivity $\varepsilon$ for spheroids of *a/b* = 1.56.

Thus, we can expect a similar effect of transverse dumbbell scattering in THz and in optics by using these materials, though the Q-factor of resonances will be lower than in microwave. Moreover, several papers have demonstrated spheroidal particle fabrication process^40–42^. The long-awaited problem in photonics of planar lasers excited laterally would be realized by metasurfaces of proposed particles. Obviously, for this frequency range, the choice of dielectric material should be corrected toward resonant values. For example, Si, Ge, GaAs particles of nanodisks and cylindric shape have shown resonant behavior in optical frequencies^43^. Moreover, polaritonic crystals NaCl, KCl, LiF, $\hbox {LiTaO}_3$ are promising for THz frequency range^44–46^. Thus, we simulate response of spheroids with *a/b* = 1.56 for different permittivities reminiscent materials from our ceramics ($\varepsilon =$ 150) to silicon in optical frequencies ($\varepsilon =$ 16). The peak of dumbbell Kerker effect still exists for low permittivities, but its Q-factor is decreased (Fig. 9). For example, for silicon ($\varepsilon =$ 16), its Q-factor is 32. Thus, we can expect that observation of transverse Kerker effect can be possible for low index materials in optics.

**Figure 10:**
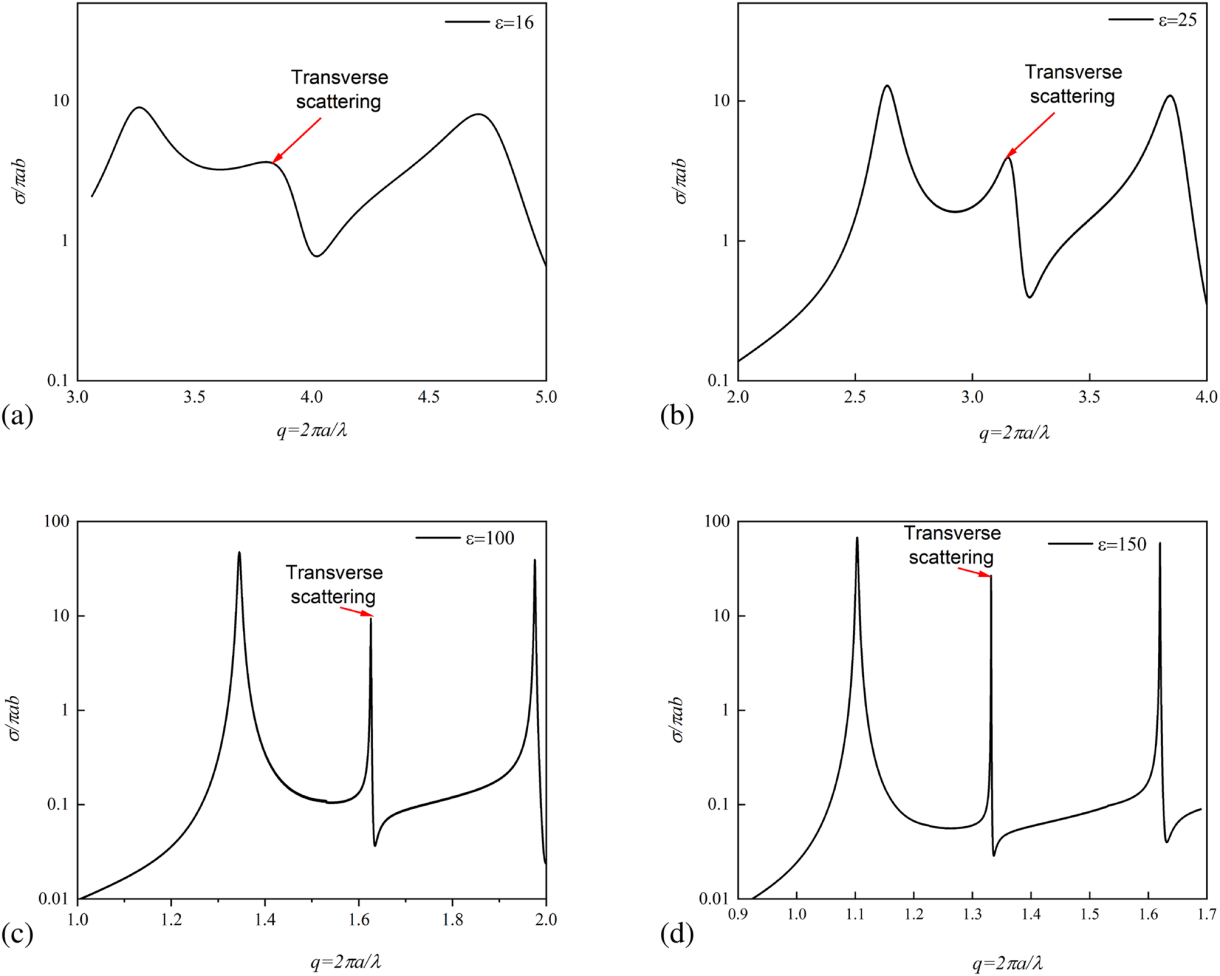
Normilized Scattering cross-section of spheroidal all-dielectric particle for different permittivities 16,25,100,150.

For this aim, we observe evolution of the transverse scattering peak dependence on permittivity and simulated scattering cross-section of spheroidal particles with size *a/b* = 1.56. For low permittivity of silicon ($\varepsilon =$ 16) the scattering cross-section is low and broadband and its maximum is 7 (Fig. 10a). To achieve the transverse Kerker effect in visible optics (red light  700 nm), the particle should have dimensions $a=423 nm$ and $b=271 nm$.

However, as the permittivity of the spheroid grows, the scattering intensity increases, and the peak associated with the transverse Kerker effect becomes narrower. One can choose the material value and dimensions according to our graphs (Fig. 10b–d). Thus, for permittivity values of 25, polaritonic crystals (NaCl, KCl, LiF) in THz regime are suitable, and for values of 100 or more, BSTO ceramics in microwaves. Meanwhile, for calculation of the Q-factors of asymmetric Fano resonance we used Fano resonance approach^47^. Thus, Q-factor obtained from the scattering spectra is $Q=\frac{f_d+f_p}{f_d-f_p}$, where the resonant region lies between the dip $f_d$ and peak $f_p$ frequencies.

## Conclusion

In this work, we proposed conditions for transverse dumbbell scattering based on interaction between multipoles of electric dipole and magnetic quadrupole moments. Our condition is beyond invisibility effects like classical and transverse Kerker effect and also anapole mode with suppressed scattering cross-section. In contrary, our scattering is enhanced in lateral directions in comparison with scattering in forward/backward directions which is suppressed. Therefore, by fabricated all-dielectric ceramic spheroidal particle based on K15U-2 capacitor ceramic, we proposed proof-of-concept microwave experiment and demonstrated dumbbell scattering in perpendicular to incident wave vector. At the same time, we observed suppressed forward and backward scattering. Our concept can be applied as ingredients for lateral excitation of planar lasers, waveguides and nanoantennas.

## Methods

The radiation of arbitrary source is formulated by electric field of multipoles. For *l* = 1, radiation is presented by electric and magnetic dipoles, while for *l* = 2 by electric and magnetic quadrupoles. Due to electric and toroidal dipoles radiate with the same radiation pattern in far-field zone, we combine them as total electric dipole ***P***

6 $$\begin{aligned}&{\mathbf{E}_{(P)} \approx \frac{\mu _{0} c^{2} }{3\sqrt{2\pi } } \frac{\exp (-ikr)}{r} \sum _{m=0,\pm 1}\left[ (k^{2} Q_{1,m} -ik^{3} T_{1,m} )\times \left( \mathbf{Y}_{1,2,m} +\sqrt{2} \mathbf{Y}_{1,0,m} \right) \right] }, \nonumber \\&\quad {\mathbf{E}_{(Qm)} \approx \frac{\mu _{0} c^{2} }{10\sqrt{6\pi } } \frac{\exp (-ikr)}{r} \sum _{m=0,\pm 1,\pm 2}\left[ -\sqrt{5} k^{3} Q_{2,m}^{(m)} \times \mathbf{Y}_{2,2,m} \right] }, \nonumber \\&\quad \mathbf{E}_{(total)} \approx \mathbf{E}_{(P)} +\mathbf{E}_{(Qm)}. \end{aligned}$$

For ${ \varphi }=\frac{\pi }{2}$ and ${\widehat{x}},{\widehat{y}},{\widehat{z}}$-basis in Cartesian coordinates

7 $$\begin{aligned} E({ \theta },{ \varphi }= & {} \pi /2)\approx \left( C_{1} P_{x} +\frac{C_{2} }{4} (4Qm_{yz} \cos 2{ \theta }+(Qm_{yy} -Qm_{xx} -3Qm_{zz} )\sin 2{ \theta })\right) {\widehat{x}} \nonumber \\&+\left( C_{1} (P_{y} \cos { \theta }-P_{z} \sin { \theta })\cos { \theta -}C_{2} (Qm_{xz} \cos { \theta }+Qm_{xy} \sin { \theta })\cos { \theta }\right) {\widehat{y}} \nonumber \\&+\left( C_{1} (P_{z} \sin { \theta }-P_{y} \cos { \theta })\sin { \theta }+C_{2} (Qm_{xz} \cos { \theta }+Qm_{xy} \sin { \theta })\sin { \theta }\right) {\widehat{z}} . \end{aligned}$$

Here $C_{1}$ and $C_{2}$ are $\theta$-independent constants:

8 $$\begin{aligned} C_{1} =\frac{e^{-ikr} c^{2} \mu _{0} k^{2} }{4\pi r} ,\ \ \ \ C_{2} =\frac{ie^{-ikr} c^{2} \mu _{0} k^{3} }{8\pi r} . \end{aligned}$$

We can obtain condition for dumbbell radiation pattern in ${\widehat{x}},{\widehat{y}},{\widehat{z}}$ directions or along ***E***, ***H***, ***k***.

It is following from Eq. 7) for $\theta$=0 and $\theta$ = $\pi$ (transverse scattering) or for $\theta$ = $\pi$/2 and $\theta$ = 3$\pi$/2 for simultaneously suppressed forward/backward scattering

9 $$\begin{aligned} E_{(total)} ({ \theta },\phi =\pi /2,r=1)\approx C_{1} P_{\alpha } +C_{2} Qm_{\beta \gamma }^{} \cos 2\theta =0 . \end{aligned}$$

We note that components $Qm{}_{xx}$, $Qm{}_{yy}$, $Qm{}_{zz}$ radiate with zero ***E***-fields for transverse scattering due to $sin2\theta$ = 0. However, function $\cos 2\theta$ for other components of magnetic quadrupole forms four-lobe radiation pattern.

Accordingly, we obtain 3 conditions for dumbbell radiation pattern along ***E***, ***H***, ***k*** vectors:

1. For radiation pattern along ***H*** vector and suppressed scattering along ***k*** and ***E***, as in our experiment:
  10 $$\begin{aligned} {C_{1} P_{x} +C_{2} Qm_{yz} =0} ,\ \ \ \ {C_{1} P_{y} -C_{2} Qm_{xz} =0} . \end{aligned}$$
  For multipoles, we have
  11 $$\begin{aligned} {\frac{P_{x} }{Qm_{yz}^{} } =-\frac{ik}{2} }, \ \ \ \ {\frac{P_{y} }{Qm_{xz}^{} } =\frac{ik}{2} } . \end{aligned}$$
2. For radiation pattern along ***k*** vector and suppressed scattering along ***E*** and ***H***:
  12 $$\begin{aligned} {C_{1} P_{x} -C_{2} Qm_{yz} =0}, \ \ \ \ {C_{1} P_{z} +C_{2} Qm_{xy} =0}. \end{aligned}$$
  For multipoles:
  13 $$\begin{aligned} {\frac{P_{x} }{Qm_{yz}^{} } =\frac{ik}{2} }, \ \ \ \ {\frac{P_{z} }{Qm_{xy}^{} } =-\frac{ik}{2} }. \end{aligned}$$
3. For radiation pattern along ***E*** vector and suppressed scattering along ***k*** and ***H***, $\varphi = 0$ :
  14 $$\begin{aligned} {C_{1} P_{y} +C_{2} Qm_{xz} =0}, \ \ \ \ {C_{1} P_{z} -C_{2} Qm_{xy} =0}. \end{aligned}$$
  For multipoles:
  15 $$\begin{aligned} {\frac{P_{y} }{Qm_{xz}^{} } =-\frac{ik}{2} }, \ \ \ \ {\frac{P_{z} }{Qm_{xy}^{} } =\frac{ik}{2} } . \end{aligned}$$
